# A One-Dollar, Disposable, Paper-Based Microfluidic Chip for Real-Time Monitoring of Sweat Rate

**DOI:** 10.3390/mi13030414

**Published:** 2022-03-06

**Authors:** Hongcheng Wang, Kai Xu, Haihao Xu, Along Huang, Zecong Fang, Yifan Zhang, Ze’en Wang, Kai Lu, Fei Wan, Zihao Bai, Qiao Wang, Linan Zhang, Liqun Wu

**Affiliations:** 1 School of Mechanical Engineering, Hangzhou Dianzi University, Hangzhou 310018, China; xukai192010065@hdu.edu.cn (K.X.); xhh761335698@hdu.edu.cn (H.X.); huangalong@hdu.edu.cn (A.H.); 202010139@hdu.edu.cn (Y.Z.); 202010053@hdu.edu.cn (Z.W.); lukai@hdu.edu.cn (K.L.); 211010006@hdu.edu.cn (F.W.); 212010101@hdu.edu.cn (Z.B.); zln@hdu.edu.cn (L.Z.); wuliqun@hdu.edu.cn (L.W.); 2 Institute of Biomedical and Health Engineering, Shenzhen Institute of Advanced Technology, Chinese Academy of Sciences, Shenzhen 518055, China; zc.fang@siat.ac.cn; 3 Shenzhen Engineering Laboratory of Single-Molecule Detection and Instrument Development, Shenzhen 518055, China; 4 School of Pharmacy, Hangzhou Medical College, Hangzhou 310059, China; wangqiao-1@163.com

**Keywords:** wearable device, microfluidic chip, sweat collecting

## Abstract

Collecting sweat and monitoring its rate is important for determining body condition and further sweat analyses, as this provides vital information about physiologic status and fitness level and could become an alternative to invasive blood tests in the future. Presented here is a one-dollar, disposable, paper-based microfluidic chip for real-time monitoring of sweat rate. The chip, pasted on any part of the skin surface, consists of a skin adhesive layer, sweat-proof layer, sweat-sensing layer, and scale layer with a disk-shape from bottom to top. The sweat-sensing layer has an impressed wax micro-channel containing pre-added chromogenic agent to show displacement by sweat, and the sweat volume can be read directly by scale lines without any electronic elements. The diameter and thickness of the complete chip are 25 mm and 0.3 mm, respectively, permitting good flexibility and compactness with the skin surface. Tests of sweat flow rate monitoring on the left forearm, forehead, and nape of the neck of volunteers doing running exercise were conducted. Average sweat rate on left forearm (1156 g·m^−2^·h^−1^) was much lower than that on the forehead (1710 g·m^−2^·h^−1^) and greater than that on the nape of the neck (998 g·m^−2^·h^−1^), in good agreement with rates measured using existing common commercial sweat collectors. The chip, as a very low-cost and convenient wearable device, has wide application prospects in real-time monitoring of sweat loss by body builders, athletes, firefighters, etc., or for further sweat analyses.

## 1. Introduction

Sweat is known to contain important information corresponding to the status of an individual’s health [1]. Sweat production is related to the stimuli underlying body thermoregulation [2]. Secretion of sweat from eccrine glands on the skin surface is an essential means of heat loss in regulating body temperature and for maintaining homeostasis [3] during heat acclimation [4]. Lost body water must be replaced to maintain normal physiologic processes; this is particularly important for body builders, athletes [5], firefighters, etc. Furthermore, sweat, like saliva and tears [6], is noninvasively induced from deeper in the body and carries a diverse array of biomolecules, ranging from small electrolytes (including Na^+^, K^+^, and Ca^2+^) and metabolites (such as glucose, lactate [7], and ethanol [8]) to hormones and larger proteins [9], which may provide vital information about physiological status and fitness level [10]. Sweat analysis could become an alternative to invasive blood tests in the future [11,12], as Heikenfeld et al. [13] demonstrated—for the first time in vivo—the complete correlation between continuous sweat data and blood data. Collecting and monitoring the rate of sweat production, which is the primary and key step for sweat research, are important for determining body condition and enabling further sweat analyses.

The whole-body wash-down method [14] is an early sweat-sampling technology and well-known as the gold standard for determining whole-body sweat loss, as all sweat runoff is collected. Subjects wearing minimal clothing ride a cycle ergometer in a plastic box. The subject, box, equipment, clothes, and all objects touched by the subject are thoroughly rinsed with deionized water to determine the whole-body sweat loss. However, this method is limited by the controlled laboratory setting, complexity of steps, and single mode of exercise testing; thus, it is not practical for field studies. Patches, composed of an absorbent material with a hydrophilic and porous structure [15], are used for regional skin surface collection and localized sweat sampling. This enables collection of sweat for hours positioned in a specific location [16] (e.g., forearm, thigh, back, or calf), but the collected sweat patch must be peeled off and carefully weighed to obtain the average sweat flow rate. The error rate is relatively high due to evaporation of sweat during the process, and it cannot show real-time flow rate data [17]. In addition, Zhang et al. [18] designed a microfluidic device with one-way-opening chambers and hydrophobic valves for sweat collection and analysis. Pan et al. [19] presented the first digital droplet flowmetry implemented on existing textile substrates for real-time flow rate measurement by counting the number of droplets. Eliot et al. [20] developed a flow rate sensor that easily couples to the outlet of a microfluidic channel to measure flow rate via periodic temporary shorting caused by droplets passing between two electrodes. The device was tested in a dynamic range as low as 25 nL·min^−1^ and as high as 9 × 10^5^ nL·min^−1^. Lindsay et al. [21] designed a skin-interfaced microfluidic system involving multilayered stacks of thin-film polymers that contain intricate microfluidic channels for personalized sweating rate and sweat chloride analytics for sports science applications.

One of the most common current commercial samplers is the Macroduct [22], which consists of a concave disk and a spiral plastic tube that collects sweat. Compared with patches, Macroducts avoid sweat leakage, contamination, and potential hydromeiosis, because the sweat is almost immediately removed from the skin. However, the device cannot be positioned on any position of the human body, so the popularity of Macroducts remains limited. A variety of modified absorbent materials, such as paper [23,24], nonwoven fabrics, textiles [25], cellulosic materials, hydrogels [26,27], and rayon pads [28] are widely used as sweat-collecting carriers. Among these materials, filter paper composed of disorderly stacked cellulose fibers with abundant hydroxyl (-OH) active groups provides high porosity, thus facilitating rapid imbibition of fluid and rendering it a very promising substrate for the immobilization of bioactive substances. Filter paper-based sweat-collecting chips are paper-based microfluidic analytical devices [29] that have generated great interest among researchers due to their portability, low cost [30], versatility, and ease of results interpretation in the analytical area due to their attractive passive movement properties (capillary phenomenon [31]) of analytes without any external forces. These chips show great promise for applications in point-of-care health systems [32], environmental monitoring [33], and food safety.

Most of the above systems, however, have complex structures or require an external collection mechanism, which is not suitable for widespread application or batch production. Paper-based microfluidic chips, which are presented in this article for the first time, can be used for real-time monitoring of sweat secretion rate. The chips, which can be pasted on any part of the skin surface, are low cost and disposable, consisting mainly of filter paper and adhesive tape with a disk shape. Sweat volume can be read directly by scale lines without any electronic elements.

## 2. Materials and Methods

### 2.1. Structure of Paper-Based Sweat Rate Monitoring Chips

**Figure 1:**
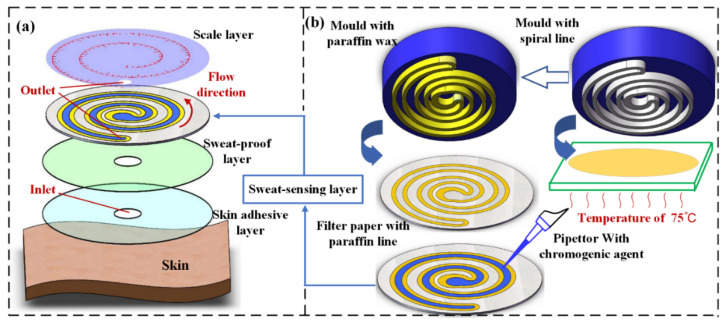
Structure of the paper-based sweat rate monitoring chip: (**a**) exploded view showing all layers of the whole chip, including the skin adhesive layer, sweat-proof layer, sweat-sensing layer, and scale layer; (**b**) method for fabrication of the sweat-sensing layer using a 3D-printed mold with a double-spiral structure.

Paper-based sweat rate monitoring chips (P-SRMCs) consist of a skin adhesive layer, a sweat-proof layer, a sweat-sensing layer, and a scale layer from the bottom to top, as shown in Figure 1a. The skin adhesive layer on the bottom, made of medical-grade double-sided adhesive tape (PICARO), is used to attach the chip to the skin surface. A small hole with a diameter of 2 mm in the center serves as the inlet for sweat secreted from the skin. The sweat-proof layer with a center hole the same size as that of the bottom layer hole is made of single-sided transparent adhesive tape (202102022207, DELE) and prevents sweat from penetrating through the double-sided adhesive material to the sweat-sensing layer and ensures that sweat flows through the center hole.

The sweat-sensing layer, as the core layer, is used to determine sweat volume. A schematic illustration of its fabrication process is shown in Figure 1b. Paraffin wax (58#, Jinmen Weijia Industro Co., Ltd., Jinmen, China) is heated using a constant-temperature heating device (ET-200, ETOOL) to the melting state of 72 °C on a section of glass slide. A mold with two parallel spiral structures 1.5 mm high is printed using a 3D printer (Pro2, Raise3D) with white polylactate as the material. The double spiral structure has a smooth flow path, can form longer channel per area than other designs, and is suitable for a disc-shape sensing chip. The parallel spiral structure is dipped into melted paraffin and then transferred onto a piece of filter paper (102, Aoke, maximum void in the range of 15–20 μm) to form paraffin wax lines (marked in yellow). The two parallel spiral wax lines constitute a micro-channel through which collected sweat travels, because the wax material is incompatible with aqueous sweat and functions as a boundary. The wax-impressed, paper-based microfluidic chip is thus one of the most promising methods for future applications because it is inexpensive, easy to use, provides rapid and robust results, and is harmless to human skin [34].

Cobalt chloride solution (AR grade, Chengdu Huaze Cobalt and Nickel Material Co., Ltd., Chengdu, China) with a mass fraction of 0.157 g/mL (0.65 mol/L), which is a good chromogenic agent to H_2_O molecules, is used as a precursor for the sweat chromogenic agent. The color of anhydrous CoCl_2_ changes from blue to red when it absorbs H_2_O molecules, forming CoCl_2_·6H_2_O. Cobalt chloride solution is harmless to human skin surface and the color of CoCl_2_·6H_2_O turns back to blue when H_2_O molecules are removed, which makes the chip possible to be reused if necessary. Cobalt chloride is slowly added to the sweat flow micro-channel using a pipettor. Then the filter paper with CoCl_2_ solution and wax lines is dried for 90 min in a vacuum drying oven (ZKXF-1, Shanghai Shuli Yiqi Yibiao Co., Ltd., Shanghai, China) set at 45 °C to remove H_2_O molecules.

The scale layer, as the top layer, is also made of single-sided transparent adhesive tape. A small hole with a diameter of 2 mm is punched and aligned to the end of the sweat flow channel in the sensing layer. The hole exposed to air is used for releasing increased air pressure caused by the entry of sweat and continuously draws sweat through the micro-channel [35]. In addition to the higher flow rate, the biggest advantage of the small hole for evaporation is that it enables convenient control of the flow rate [36]. By changing the size of the small hole, the flow rate can be easily regulated. The scale, made of red stamp ink (YY01, GSD), is impressed on the reverse side using a mold to avoid the possibility of being removed while the subject is exercising.

The diameter of all four above-mentioned layers is 25 mm and cut by a Laser Cutting Machine (3020, KETAILASER). A piece of P-SRMC is prepared according to the process shown in Figure 1, and the edge of the assembled chip is covered with paraffin wax to prevent external water molecules from affecting the test results. To make subjects more comfortable while doing exercise, the overall thickness of the microfluidic patch was limited to 0.3 mm to enable good flexibility and good compactness against the skin surface.

### 2.2. Sweat Micro-Channel Parameters

**Figure 2:**
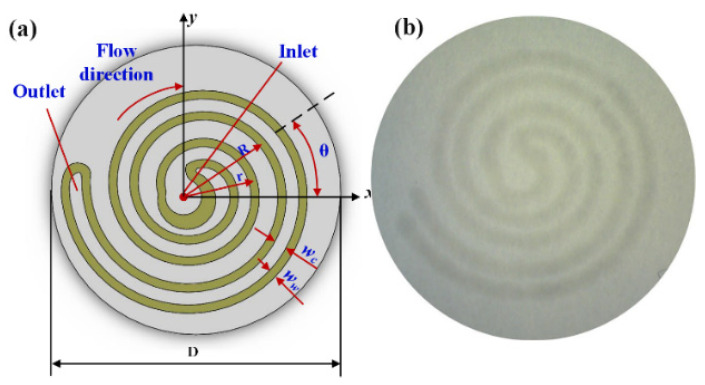
Sweat channel with double spiral structure: (**a**) structure sketch and (**b**) photomicrograph of the wax channel impressed on filter paper using a mold.

The ratio of the maximum volume of sweat monitored to the size of the whole chip is the most important index for a wearable device. The whole chip size is determined by the width of the wax line (*w*_w_) and the sweat channel (*w*_c_). If the wax line is too narrow, sweat will leak out across it in the filter paper and affect the measured result; thus, a sweat-leaking experiment was conducted to determine the minimum wax width. As to the sweat channel, if the channel is too narrow, the sweat travel velocity will be too slow. Thus, a sweat flow velocity experiment was also conducted to determine the minimum flow channel width. A chamfered fillet structure was applied on the inlet and outlet parts. To minimize the chip size, the shape of the microchannel was designed as a double spiral structure, as shown in Figure 2. In Cartesian coordinates, the equations of two spiral lines on external and internal sides are respectively obtained as:

(1) $$\left\{ \begin{array}{l} r=a+b(\theta /2\pi )\\ x=r\cos\theta \\ y=r\sin\theta \end{array}\right.$$

(2) $$\left\{ \begin{array}{l} R=a+w_{w}+w_{c}+b(\theta /2\pi )\\ x=R\cos\theta \\ y=R\sin\theta \end{array}\right.$$

where *r* is the radius of the spiral line on the external side, *R* is that on the internal side, and *θ* is the angle of spiral lines. The differential of *R* and *r* is (*w*_w_ + *w*_c_).

### 2.3. Chip Assembly

**Figure 3:**
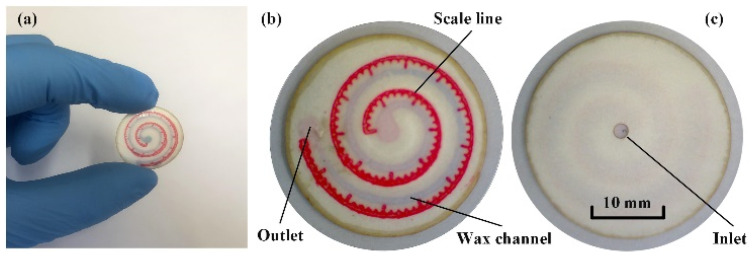
(**a**) Photograph of a P-SRMC chip, (**b**) top view, and (**c**) bottom view of the assembled chip.

A complete chip fabricated according to the above process is shown in Figure 3. All the assembly steps are conducted under a microscope (3R-MSUSB401, Anyty). Scale lines should be located parallel to and on the lateral side of the wax channel to show the displacement of collected sweat traveling through the channel. The scale spacing is 1 mm, with a range of 0–90 mm. The edge of the chip appears grey in color because all the layers are shaped by a laser cutting machine, and the edge is burned to ashes. This does not affect the chip’s ability to collect sweat because the whole edge is covered by paraffin wax to prevent external water molecules from affecting the test results. The assembled chip is dried for 30 min in a vacuum drying oven to thoroughly remove water molecules.

### 2.4. Chip Calibration

As a type of measuring device, the chip must be calibrated before being tested on the human body. Sweat, drawn from human skin using a suction tube, is added to the chip through the inlet in the scale layer using a pipettor (volume resolution of 0.1 μL). The liquid is added drop by drop with a single drop volume of 1.0 μL. Each droplet should be added after the previous droplet has thoroughly infiltrated into the wax channel by observing the inlet area under a physical microscope. The displacement caused by sweat moving forward along the helix wax channel is recorded via the scale lines after each sweat droplet is added based on the channel with sweat molecules turning from blue to red.

### 2.5. Testing on Human Volunteers

**Figure 4:**
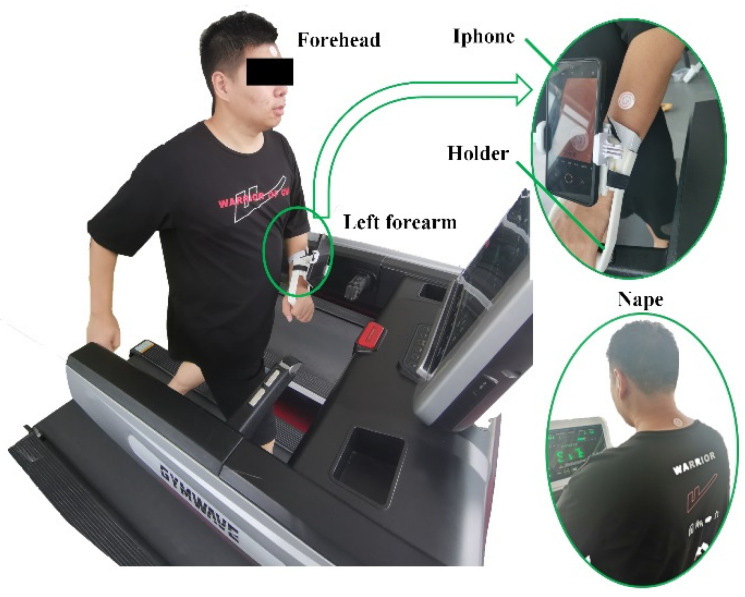
Experimental setup involving a male volunteer running under controlled conditions with chips located on the forehead, left forearm, and nape of the neck.

Two healthy, active male and female volunteers, 24 years old, participated in indoor running sweat-collecting trials. The weight and height of male candidate are 83 kg and 175 cm, respectively and that of female are 52 kg and 162 cm, respectively. The average room temperature and humidity were 28.9 °C and 74%, respectively. The volunteers ran on a treadmill (SH-T5170) under controlled conditions with a running speed of 8.8 kph. When considering localized areas of interest, the choice of sampling area is very important, as it has been reported that sweat rate depends significantly on the sampling location [37]. The sweat-secreting rates on the forehead, nape of the neck, and left forearm are larger than the others on human body. These positions are usually exposed to air while candidates are exercising and suitable for affixing the P-SRMCs. In this study, the chips were positioned on the forehead, nape of the neck, and left forearm, as shown in Figure 4.

A mobile phone (iPhone XR with resolution of 1080p and frame rate of 240 fps), fixed on the left forearm by a designed holder and with its camera aligned with the sensing chip, was used to record video of the sweat-collection process. The displacement in chips on the forehead and nape of the neck were recorded by photos taken every 5 min and then used to calculate sweat volume using Formula (3), as the displacement value can be read from the scale lines. Scientific research shows that it takes about 30 min for an average person to have the best sports effect. So, the testing time is set as slightly longer than 30 min. Sweat rates (υ_s_) are expressed as g·m^−2^·h^−1^, for it is usually calculated in grams per square meter of body surface area per hour.

## 3. Results and Discussion

### 3.1. Sweat Micro-Channel Parameters

**Figure 5:**
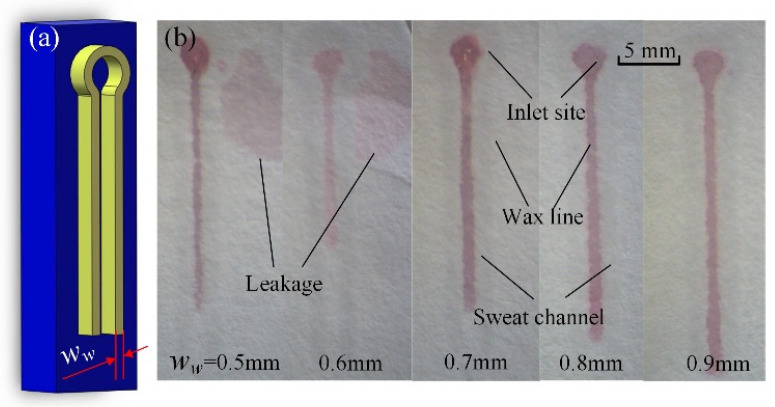
Sweat leakage experiment between neighboring wax lines with different widths: (**a**) mould for wax channels; (**b**) photo of channels with sweat.

A sweat leakage experiment was conducted to determine the minimum wax line width to prevent sweat leaking through the filter paper. 3D-printed molds of different sizes were designed to form wax lines with different widths on the filter paper. Sweat (2.5 μL) was added on the initial site using a pipettor, and the paper was placed on a piece of horizontally situated glass slide to observe leakage using a microscope. As shown in Figure 5, when the width (*w_w_*) of the wax lines was 0.5 mm and 0.6 mm, the CoCl_2_ solution leaked across the wax lines. When the width was no less than 0.7 mm, the wax lines completely prevented leakage. Therefore, 0.7 mm was determined to be the minimum wax width.

The sweat channel width (*w_c_*) is another important parameter for sweat collection, as it determines the travel rate of the sweat. Although Darcy’s law, Lucas-Washburn equation [38], Modifications to Darcy’s law and LW equation [39], Richards equation [40], and flow simulation or visualization tools [41] can approximately calculate the micro-fluid flow behavior in paper material, fluid imbibition into paper is a complex process governed by a highly coupled system of length and time-scaled parameters. Therefore, the sweat channel width in filter paper is typically determined by experiment, traditionally called a trial-and-error strategy. Besides that, capillary flow velocity inner filter paper is determined by pore radius of the porous media in the hydrophilic channel and the size of the channel according to Ref. [42]. The shape of channel bas little effect on flow velocity of sweat if the flow rate is relatively low. The experiment result in straight channel is suitable for spiral-shape ones.

**Figure 6:**
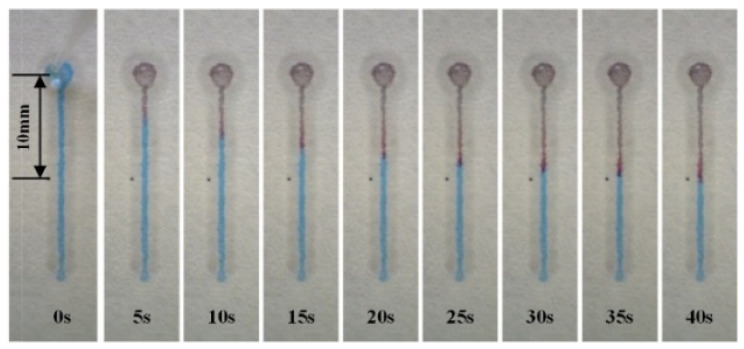
Recordings illustrating the process of sweat flow in a paper-based wax channel with a width of 1.2 mm and displacement of 10 mm.

**Figure 7:**
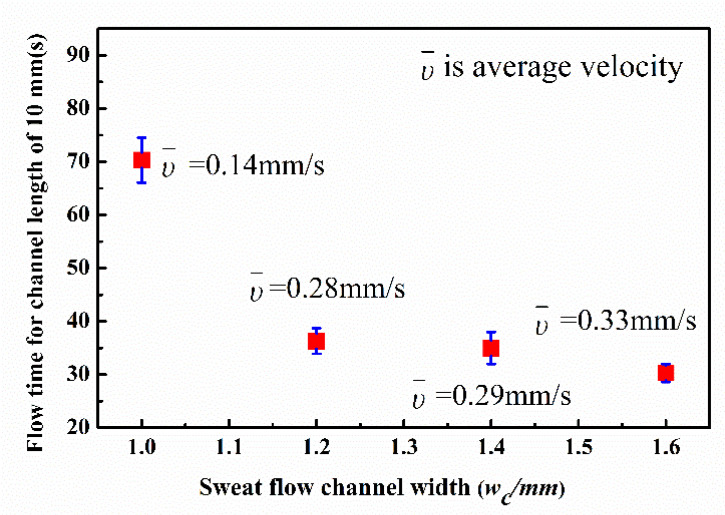
Variation in sweat flow velocity with width of the paper-based channel.

To minimize the chip size, a width of 1.2 mm was chosen as the micro-channel parameter for the final sweat rate monitoring chip. A CoCl_2_ solution with a concentration of 0.65 mol/L and total volume of 1.6 μL was deposited in the channel for result visualization. The flow distance was set at 10 mm to calculate the average flow velocity, $\bar{\upsilon }$. The process of sweat flow in the paper-based wax channel with a width of 1.2 mm is shown in Figure 6 and Video S1 in the Supplementary Material. Figure 7 shows the influence of channel width on sweat flow velocity in straight paper-based channels. The average flow velocity increased rapidly in channel widths set at 1.2 mm, 1.4 mm, and 1.6 mm. More than 70 s (average velocity of approximately 0.14 mm/s) was required for sweat to travel along the channel with a relatively narrow width of 1.0 mm.

**Figure 8:**
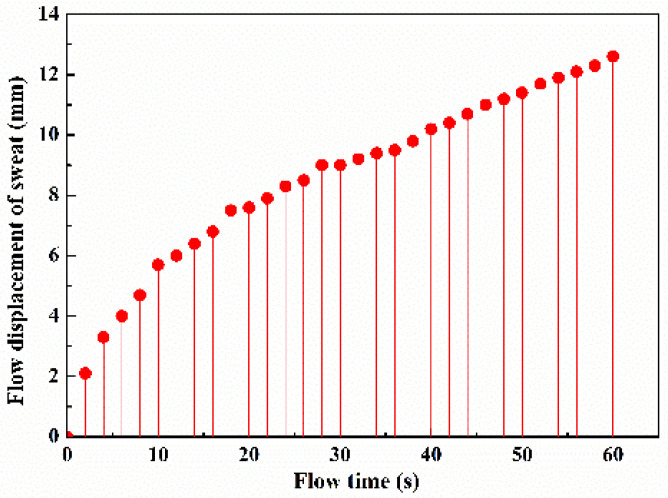
Record of sweat flow displacement in a paper-based wax channel with a width of 1.2 mm.

Figure 7 shows average sweat travel velocity data. However, the flow velocity was not constant and decreased slowly because of the increasing flow resistance downstream in the micro-channel. Therefore, flow displacement in a paper-based wax channel with a width of 1.2 mm was recorded at different times, and the results are shown in Figure 8. The velocity was relatively high near the sweat inlet site then decreased until displacement was less than approximately 7 mm and finally became approximately constant. The reason the velocity decreased in the first section is that CoCl_2_ crystal particles were scoured downstream by sweat flow, causing an enrichment that increased the flow resistance. The experiment results show that sweat with a volume of 2.5 μL filled the paper-based channel in approximately 60 s.

### 3.2. Chip Calibration Results

**Figure 9:**
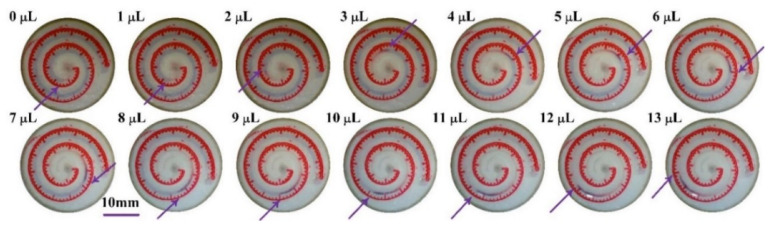
Calibration of a P-SRMC.

In chip calibration experiments, it is time-consuming to completely fill the wax channel with sweat drop by drop. A record of sweat traveling through a certain piece of P-SRMC chip during the calibration process is shown in Figure 9. The color of the channel through which the sweat travels changed from blue to red. The scale in red shows the displacement of sweat as it travels. As shown in Video S2 in the Supplementary Material, it takes each 1-μL sweat droplet approximately 5 min on average to infiltrate the wax channel completely. The time required by latter droplets is longer than that of former droplets because of the gradually increasing flow resistance as drops are added.

**Figure 10:**
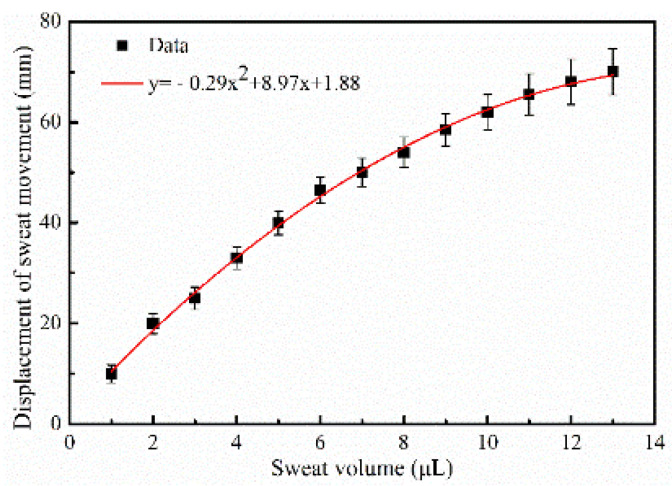
Second-order fitting curve for the variation in displacement with added sweat volume determined using scale lines.

Ten samples of sweat colleting chips were tested to obtain an average result and determine their consistency. As shown in Figure 10, the displacement of sweat moving forward along the spiral wax channel was recorded using the scale lines after different volumes of sweat were added from the chip inlet.

A second-order fitting, as is shown in Formula (3), was carried out on the variation:

(3) $$y=-0.29x^{2}+8.97x+1.88\;\left(0<x<13.4\right)$$

where *y* is displacement (mm) and *x* is the volume of sweat (μL). The relative variance was ~10%.

According to the experimental result, the maximum volume of sweat collected in a single chip is above 14 μL, and the sensitivity (s) can be calculated by:

(4) $$s=\frac{dy}{dx}=-0.58x+8.97$$

The sensitivity decreases gradually with increasing volume of sweat collected. Figure 10 shows that it reaches a maximum value of 8.97 mm·μL^−1^.

### 3.3. Testing Results Using Human Volunteers

**Figure 11:**
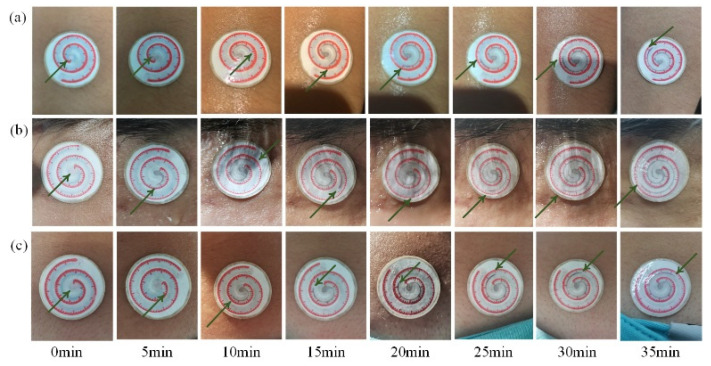
Photographs of P-SRMC changing color on the (**a**) left forearm, (**b**) forehead, and (**c**) nape of the neck taken every 5 min while the volunteers was running on a treadmill.

**Figure 12:**
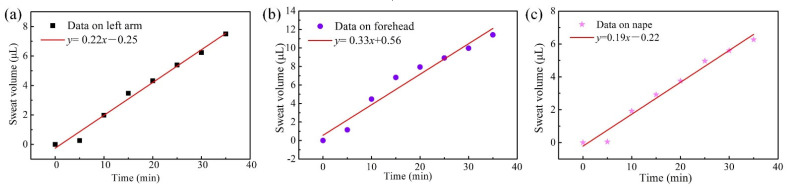
Flow rate of sweat collected from (**a**) the left forearm, (**b**) forehead, and (**c**) nape of the neck measured using the P-SRMC.

The displacement caused by sweat moving forward was read using the scale lines on the sweat-collecting chips locating on the left forearm, forehead, and nape of the neck of the human volunteers, as shown in Table 1. The sweat secretion velocity on male candidate is faster than that on female candidate. Photographs of P-SRMC changing color on male skin surface are shown in Figure 11. The time interval for the recording was 5 min. Video of sweat traveling through the chip on the left forearm of male candidate was recorded using a mobile phone, as shown in Video S3 in the Supplementary Material. The values were consequently converted to volume of sweat collected using Formula (3). According to the shape of the above fitting function, the smaller of the two solutions for the unitary quadratic equation is the sweat volume. The conversion results for each displacement of sweat traveling through the chip are shown in Figure 12.

The test results showed that the relationship between volume of sweat secreted from the left forearm, forehead, and nape of the neck and running time was approximately linear. In other words, the sweat secretion velocity is approximately uniform. Considering that the diameter of the sweat-collecting inlet is 2 mm, the flow rate per area can be calculated. However, during the experiment, some sweat is converted into vapor around the chip, as the temperature of the skin surface at the measurement site is much higher than that of the ambient environment. The sweat vapor formed close to the inlet hole will seep into the sensing layer and cause the experimental result to be greater than the actual value. To minimize this effect, the correction coefficient *δ* was calculated for the collecting area and set as 2. Therefore, the collecting area (*A*_c_ = 4π mm^2^) is twice that of the inlet hole diameter.

Among the three measured sites, the forehead had the highest average sweat secreting velocity (*V_f_*), 0.33 μL·min^−1^, which is the slope of the fitted straight line in Figure 12b. The flow velocity per area for the forehead position was calculated using Formula (5) and equaled 0.026 μL·mm^−2^·min^−1^.

(5) $$R_{f}=\frac{V_{f}}{A_{c}}$$

Sweat rate is usually calculated in grams per square meter of body surface area per hour. Therefore, the rate of sweat secretion from the forehead position was 1734 g·m^−2^·h^−1^ if we assume that the density of sweat is 1.1 g·cm^−3^. The test result showed good agreement in order of magnitude with that measured using absorbent pads [43] and Macroduct [22], which are common commercial sweat collectors for determining sweating rate. As shown in Figure 12a,c, the sweat secretion velocity on the left forearm (*V*_arm_) and nape of the neck (*V*_nape_) was 0.22 μL·min^−1^ and 0.19 μL·min^−1^, respectively. The secretion rates for the above two positions were 1156 g· m^−2^·h^−1^ and 998 g·m^−2^·h^−1^, respectively. Therefore, the sweat rate of the left forearm was much lower than that of the forehead and greater than that of the nape of the neck for regional variations in human eccrine sweat gland density and local sweat secretion rates during the thermal loading in exercising individuals [44].

## 4. Conclusions

This paper describes a low-cost, disposable, paper-based microfluidic chip for real-time sweat secretion rate monitoring. The chip consists primarily of a skin adhesive layer, a sweat-proof layer, a sweat-sensing layer, and a scale layer with a disk-shape from the bottom to top. Double spiral wax lines impressed in a piece of filter paper using a mold serve as the micro-channel through which the collected sweat travels. The micro-channel is pre-filled with a chromogenic agent to show displacement of the sweat as it travels, so sweat volume can be read directly using scale lines without any electronic elements. The diameter and thickness of the whole chip are 25 mm and 0.3 mm, respectively, which allows for good flexibility and good compactness with the skin surface. Tests of sweat flow rate monitoring on the left forearm, forehead, and nape of the neck of human volunteers during running were conducted. The average sweat secretion rates of the three positions were 1156 g·m^−2^·h^−1^, 1710 g·m^−2^·h^−1^, and 998 g·m^−2^·h^−1^, respectively, in good agreement with values measured using existing common commercial sweat collectors. The P-SRMC chip, as a very low-cost, disposable, and easily fabricated wearable device, has a wide range of potential applications in real-time monitoring of sweat loss for body builders, athletes, firefighters, etc., or for further sweat analyses.

## Figures and Tables

**Table 1:** Record of test results for human volunteers.

Time/min	Left Forearm/mm		Forehead/mm		Nape of Neck/mm	
	Male	Female	Mail	Female	Mail	Female
0	0	0	0	0	0	0
5	4.2	3.1	11.8	6.3	3.5	2.6
10	18.5	15.0	36.8	20.7	19.2	14.6
15	29.5	20.1	49.5	31.3	26.8	19.5
20	35.2	25.3	54.8	42.9	32.6	21.9
25	41.8	30.0	58.8	49.8	40.5	29.8
30	46.5	38.2	61.5	52.1	44.2	34.2
35	52.8	45.8	66.5	56.5	47.9	38.6

## Data Availability

The data presented in this study are available from the corresponding author, H.W, upon reasonable request.

## Supplementary Materials

The following are available online at https://www.mdpi.com/article/10.3390/mi13030414/s1, Video S1: Sweat flow process in paper-based channel with width of 1.2 mm; Video S2: Calibration of sweat rate real-time monitoring chip; Video S3: Test of sweat flow rate monitoring on left forearm of a male volunteer doing running exercise.

## Nomenclature

*r*	radius of spiral line on the external side, mm	*s*	sensitivity of P-SRMC sensor, mm·μL^−1^
*R*	radius of spiral line on the internal side, mm	*x*	volume of secreting sweat, μL
*w_w_*	width of the wax line, mm	*y*	is displacement, mm
*w_c_*	width of the sweat channel, mm	*A_c_*	sweat collecting area, mm^2^
*θ*	angle of spiral lines, rad	*V_f_*	sweat secreting volume velocity from forehead, μL·min^−1^
$\bar{\upsilon }$	sweat average flow velocity in wax channel, mm/s	*V*_arm_	sweat secreting volume velocity from left forearm, μL·min^−1^
υ_s_	sweat secreting rate, g·m^−S2^·h^−1^	*V*_nape_	sweat secreting volume velocity from nape of the neck, μL·min^−1^
